# Categorical grouping is not required for guided conjunction search

**DOI:** 10.1167/jov.20.8.30

**Published:** 2020-08-28

**Authors:** Igor S. Utochkin, Vladislav A. Khvostov, Jeremy M. Wolfe

**Affiliations:** HSE University, Moscow, Russia; Visual Attention Laboratory, Brigham & Women's Hospital, Cambridge, MA, USA; Harvard Medical School, Boston, MA, USA

**Keywords:** visual search, conjunction search, top-down guidance, Guided Search, categorization, grouping, segmentation, ensemble statistics

## Abstract

Knowledge of target features can guide attention in many conjunction searches in a top-down manner. For example, in search of a red vertical line among blue vertical and red horizontal lines, observers can guide attention toward all red items and all vertical items. In typical conjunction searches, distractors often form perceptually vivid, categorical groups of identical objects. This could favor the efficient search via guidance of attention to these “segmentable” groups. Can attention be guided if the distractors are not neatly segmentable (e.g., if colors vary continuously from red through purple to blue)? We tested search for conjunctions of color × orientation (Experiments 1, 3, 4, 5) or length × orientation (Experiment 2). In segmentable conditions, distractors could form two clear groups (e.g., blue steep and red flat). In non-segmentable conditions, distractors varied smoothly from red to blue and/or steep to flat; thus, discouraging grouping and increasing overall heterogeneity. We found that the efficiency of conjunction search was reasonably high and unaffected by segmentability. The same lack of segmentability had a detrimental effect on feature search (Experiment 4) and on conjunction search, if target information was limited to one feature (e.g., find the odd item in the red set, “subset search,” Experiment 3). Guidance in conjunction search may not require grouping and segmentation cues that are very important in other tasks like texture discrimination. Our results support an idea of simultaneous, parallel top-down guidance by multiple features and argue against models suggesting sequential guidance by each feature in turn.

## Introduction

Our visual environment consists of many objects differing in a number of features (such as color, size, shape, texture, etc.). Some objects can have unique features not shared by any other objects, whereas other objects can share at least some features with some of the other objects. When we search for something having a unique feature (e.g., a red item among many green items), we perform a feature search that is usually very easy if the target is sufficiently different from distractors (Egeth, Jonides, & Wall, 1972; Treisman & Gormican, 1988; Wolfe & Horowitz, 2017). But if our target is an item sharing features with many other items and differing only in how these features are combined together (e.g., a red vertical thing among many red horizontal and green vertical things), we perform a conjunction search that is typically more difficult (Treisman & Gelade, 1980). A crucial difference between behavioral patterns observed in feature and conjunction search is that the search time does not change very much with the number of items (set size) in the former case and increases almost linearly with the set size in the latter case. Treisman's classic Feature Integration Theory (Treisman & Gelade, 1980) proposed that feature searches can be performed in parallel by unlimited-capacity preattentive mechanisms while conjunction searches require the serial deployment of attention to each location (at a rate ∼20–40 ms/item) until the target is found or its absence is confirmed. The serial deployment of attention is supposed to permit the correct binding of features into a coherent, updatable “object file” in working memory and, thus, enable conscious object recognition (Kahneman, Treisman & Gibbs, 1992; Rensink, 2000; Treisman & Gelade, 1980; Wolfe, Võ, Evans & Greene, 2011).

This classical pattern of slow, serial conjunction search has been challenged by many experiments (e.g. Egeth, Virzi & Garbart, 1984; Enns & Rensink, 1990; Nakayama & Silverman, 1986; Wolfe, Cave & Franzel, 1989). For many conjunctions, the slopes of the Reaction Time (RT) × Set size function are shallower than those predicted by the standard item-by-item serial search. Many conjunction searches produce slopes that are intermediate between very efficient searches with slopes of the RT functions close to 0 ms/item and inefficient searches such as search for the letter T among Ls (20–35 ms/item). This led researchers to an idea that the visual system might use shortcut strategies making otherwise serial conjunction search more efficient.

One, intuitively appealing possibility is that conjunction search could be made more efficient by a two-step process. First, attention would be limited to a subset defined by one, specific feature. Second, search would then proceed through that subset. In a clear example of subset search, Egeth et al. (1984) had observers search for a letter target. If the color of the letter was known, the data clearly showed that observers could restrict search to the group of items of that color. If the target is defined by the conjunction of two basic features, then the second step, the search through the subset can become very close to the efficient feature search (Friedman-Hill & Wolfe, 1995; Huang & Pashler, 2007) or at least can exploit parallel processing (Pashler, 1987; Zohary & Hochstein, 1989). For example, an efficient search for a red vertical target might occur when observers first select the red subset and then perform an efficient search for vertical within that subset. We will refer to this class of theories as *sequential models*. Alternatively, *simultaneous models*, such as Wolfe's Guided Search model (Wolfe, 1994; Wolfe, 2007; Wolfe et al., 1989) propose that several features can be used at the same time to guide the deployment of attention towards target conjunctions. Thus, in that search for a red vertical target, attention could be guided to red and to vertical items at the same time, with any red vertical target getting a double dose of guidance and becoming a high-priority candidate for drawing attention. The more target features are known, the more efficiently they can guide conjunction search (Nordfang & Wolfe, 2014; Wolfe et al., 1989). Other theories emphasize the role of grouping by proximity and similarity (e.g. Bacon & Egeth, 1994; Duncan & Humphreys, 1989; Humphreys & Muller, 1993) that transform otherwise separate items into larger chunks that can be attended simultaneously, thus increasing the search rate. These chunks can be weighted by their similarity with the top-down target template, so that most target-similar items or groups of items are attended first, thus increasing search efficiency.

The success of any such shortcut strategy seems to depend on an ability to define which items have relevant features and which items do not. It appears trivial in a standard, laboratory conjunction search. When you look for a red vertical line among red horizontal and blue vertical lines in a standard experimental display, the colors and orientations are usually very well-defined. All red lines are usually exactly the same shade of red, and all blue lines are the same blue, forming two color groups. Similarly, in orientation, there would be clear vertical and horizontal groups. Thus, in this standard conjunction search, there would be clear groups of red horizontal and blue vertical items along with a possible red vertical target. However, such a simple situation is not always typical of searches in the real world. For example, imagine that you are looking for a small red volume on a bookshelf filled with big red and small blue volumes. The features “big,” “small,” “red,” and “blue” might be much less homogeneous. Distractor heterogeneity generally makes search less efficient (Duncan & Humphreys, 1989). More importantly for our current research question, growing heterogeneity also diminishes the *categorical* distinctiveness of the target and distractor features. There might be a range of reddish and bluish colors whereas the sizes might vary continuously from big to small. How does guidance proceed when features are variable and do not form clear groups or categories? For example, if sizes of books are heterogeneous, how would one define where the subset of large books ends and where the small subset begins? This problem can be especially important for theories assuming the binary character of feature representations controlling visual selection (e.g., Huang & Pashler, 2007). When distractors do not form categorically distinct groups, can attention be efficiently guided toward target features in conjunction search?

## The role of categorical grouping in visual search

Previous work shows that categorization can play an important role in guiding attention in feature searches. For example, Wolfe, Friedman-Hill, Stewart, and O'Connell (1992) demonstrated this in a search for orientation targets. They reported that, in a search among heterogeneous distractor orientations, it is easy to find a target if this target is the only item belonging to a coarse category, such as “steep,” “shallow,” “right tilt,” or “left tilt.” On the contrary, it is harder to find the target if it shares categorical features with the distractors (e.g., a steep target tilted to the right among steep distractors tilted to the left and flat distractors tilted to the right). Utochkin and Yurevich (2016) probed the boundary conditions of category formation in feature search for size and orientation targets. They manipulated the statistics of the features in distractor distributions within a fixed range. “Segmentable” displays were composed of lines of just a few, widely spaced values in the feature dimension (e.g., 0°-, 22°-, and 45°-oriented lines). Non-segmentable displays had lines more smoothly distributed over the same range of values (e.g., 0°-, 5°-, 10°-, 15°-…, 45°-oriented lines). A target stimulus, if present, was always tilted away from the orientations of the distractors (e.g., 135°) so that it was always categorically distinct and thus all manipulations concerned grouping or segmentation between distractors only.

**Figure 1.**
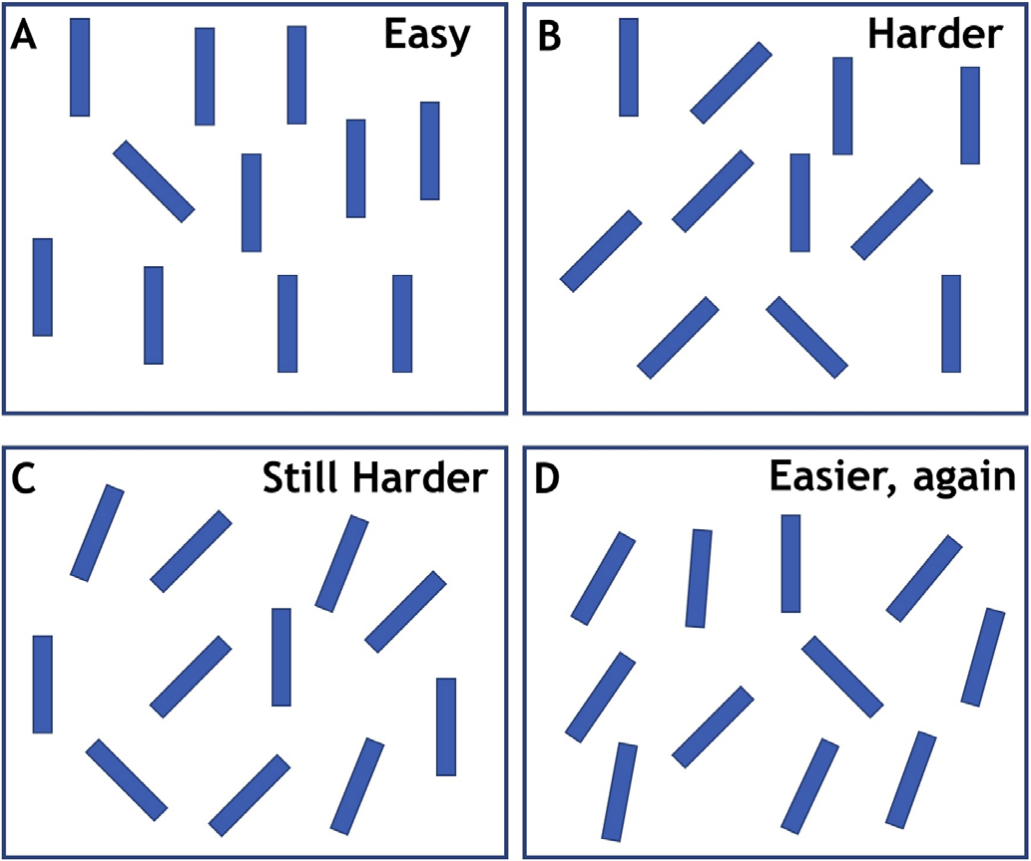
Search for a left tilted item becomes more difficult as distractors become more heterogeneous (A–C) until the distribution of distractors becomes relatively smooth (D), at which point, search becomes easier.

As illustrated in Figure 1, Utochkin and Yurevich (2016) found that the speed of search was non-monotonically related to the smoothness of distractor distribution. The addition of feature values slowed down the search as long as the distribution remained segmentable. Thus, search for 135° among 0° and 45° (Figure 1B) was slower than search among 0° alone or 45° alone (Figure 1A), and search among 0°, 22°, and 45° (Figure 1C) was slower than search among 0° and 45°. However, when the distribution became non-segmentable (Figure 1D: 0°, 5°, 10°…45°), then search became faster than in the sharply distributed distractors, although, by some measures, heterogeneity reached its maximum in the non-segmentable distribution. Utochkin and Yurevich (2016) concluded that this nonmonotonic pattern might be related to the categorical grouping of distractors. In the segmentable distributions, the differences between any two neighboring values in the feature space were large enough so that items of the same orientation could form discrete groups. Each of those groups might attract attention and might need to be rejected as non-targets in a serial manner. When the distribution is non-segmentable, differences between orientations can fall below the threshold for “preattentive” grouping of orientations (e.g., Foster & Ward, 1991). If the distractors form a single group, even if there is variability in the group, it may be possible to reject the entire group in one, efficient step. Overall, the concept of “segmentability” refers to the shape of a feature distribution (whether it has one or several peaks). Variations in segmentability lead to the perception of arrays of multiple objects as consisting of a single group or of several categorical groups (see Utochkin, 2015, for a general framework). Note that the term “segmentability,” as we use it here, is not strictly dependent on spatial proximity. Rather, it reflects an ability to perceive any set of items as consisting of elements of one or several kinds, even if they are spatially intermixed. For example, one can see a set of apples among a set of leaves, although the apples and the leaves do not form spatially separate groups or surfaces with a single boundary between them).

The findings showing the role of categorical differences in feature search (Utochkin & Yurevich, 2016; Wolfe et al., 1992) combined with some studies of conjunction search (Anderson, Heinke & Humphreys, 2012; Driver, McLeod & Dienes, 1992; Grossberg, Mingolla & Ross, 1994; Pashler, 1987; Wang, Kristjansson & Nakayama, 2005; Zohary & Hochstein, 1989) suggest that segmentability might influence conjunction search as well. Investigating the role of segmentability in conjunction search is the purpose of this study.

## Overview of experiments

As noted above, the typical laboratory conjunction search task (like search for a red vertical line among red horizontal and blue vertical distractors) makes use of highly segmentable features that form neat groups, each sharing one of the properties of the target very precisely (a red horizontal group and a blue vertical group, in this case). The presence of these groups might provide favorable conditions for guiding attention by *well-defined* feature categories. By contrast, it might be thought that smooth, non-segmentable feature distributions would make conjunction search more difficult. Such displays make the distractors more heterogeneous. Moreover, they make the groups of distractors ill-defined. In the standard display, it would be easy to decide if an item was red or blue. In this example, a non-segmentable display would contain purple items that blur the red-blue boundary. These circumstances might make feature guidance more complicated and the conjunction search less efficient. The present work shows that guided conjunction search is relatively immune to this manipulation.

We report a series of five experiments testing the role of feature segmentability in guided conjunction search. In Experiments 1 and 2, observers looked for a color-orientation or length-orientation conjunction in displays where segmentability was manipulated by changing the distributions of both relevant feature dimensions in the distractors. Our main result is that conjunction search remains quite efficient and is largely unaffected by feature segmentability. To account for this interesting and seemingly counterintuitive result, we propose that the visual system uses information about two target features to guide top-down attention in a manner that does not depend on grouping or segmentation per se (see Interim discussion). This suggestion is tested in detail in Experiments 3 and 4. In Experiment 3, we test whether feature segmentability affects search in conjunction displays when top-down guidance is limited to only one of the two features of the target. This is a version of the “subset search” task of Friedman-Hill and Wolfe (1995) in which participants were instructed to look for an item with an unknown odd orientation in a subset of items with a predefined color (this task artificially increased sequential account of the search). Our results show that, whereas the lack of segmentability increases the overall search time (presumably by additional time required to figure out which orientation is assigned to the target), it does not affect the search efficiency of the search stage when the target orientation is determined and can be added to the target color information. In Experiment 4, we demonstrate that conjunction search can be quite efficient for non-segmentable stimuli even if exactly the same non-segmentable feature distributions produce slow feature searches. This is a rare case where a manipulation that degrades feature search (find “red”) does not degrade conjunction search (find “steep red”) because conjunction search can benefit from simultaneous guidance by two features. Experiment 5 was a replication of Experiment 1 where we doubled the sample size and extended the number of tested set sizes. It shows that the lack of the segmentability effect on conjunction search is robust.

## Experiment 1

In Experiment 1, observers searched for a specific color-orientation conjunction (e.g., a red steep line) among colored line distractors that could resemble the target in color or orientation but not both. Our principal manipulation concerned the distributions of colors and orientations in the distractors. In the segmentable condition, the features were the extreme points of the feature ranges used for the experiment. Colors were blue or red hue. Orientations were very steep (meaning, 10° where 0 is vertical) or very flat (80°). For non-segmentable distributions, the feature values were drawn uniformly from the same ranges, so that distractors included the extremes as well as the intermediate features (i.e., various shades of reddish, bluish and purplish, or various orientations from very flat to very steep). We manipulated the segmentability of colors and orientations orthogonally to be capable to estimate the relative contribution of each feature dimension into segmentation-driven conjunction search (if any).

### Method

#### Participants

Twelve volunteer observers were recruited from the Cambridge, MA community (8 female, mean age – 28.1). Sample size for Experiments 1 to 4 was determined by the data set from Wolfe, Palmer, and Horowitz (2010), in which the standard deviation (*SD*) of slopes is about 0.3 of the mean slope and *SD* of RTs is about 0.4 of the mean RT. To detect a doubling of the RT x set size slope and/or a 100 ms main effect in RT, G*Power 3.1 toolbox (Faul, Erdfeler, Lang, & Buchner, 2007) showed that 11 observers were required to achieve power (1 − β) = 0.8 with Type I error (*α*) = 0.01. All participants had normal visual acuity and were found not to have any color vision deficiency as assessed by the Ishihara color blindness test. They also reported having no neurological problems. At the beginning of experiment, they gave written informed consent as approved by the Institutional Review Board of Brigham and Women's Hospital.

#### Apparatus and stimuli

Stimulation was developed and presented using PsychoPy (Peirce et al., 2019). Stimuli were presented on iMac A1225 (EMC 2211) with a refresh frequency of 60 Hz and a 1920- × 1200-pixel spatial resolution. A 24.21° × 24.21° square at the center of the screen was used as the “working” field for presenting stimuli; the remaining screen space remained black. The working field was divided into 5 × 5 = 25 cells by an imaginary grid (each cell side was 4.91°). Each cell could be used as the location for a single line element of the display (some cells could be empty in a particular trial). Within the cell, lines were randomly jittered within a ±0.82° range in both horizontal and vertical directions. The cell in which the target appeared (if present) was chosen randomly on each trial, providing a globally uniform spatial distribution of the target during the experiment.

Each search array contained sets of lines of various colors and orientations. Lines were 0.16° wide and 2.05° long. The orientations were drawn from a set ranging between 10° and 80° in steps of 10° (eight orientations). The colors were drawn from a hue distribution along the psychophysically uniform CIE Lab color wheel; the distribution ranged from 270° (blue) to 360° (red) in steps of 12-13° on the hue circle between neighboring colors (thus, eight colors). The use of CIE Lab color wheel allowed us to manipulate only hue, while brightness and saturation remained constant. For segmentable feature distributions, only extreme values could be presented (10° and 80° for orientation, 270° and 360° for color), each feature value was shared by 1/2 of distractors. For non-segmentable feature distributions, the whole range of eight values was presented providing a smooth distribution from one extreme to another, each feature value was shared by 1/8 of distractors (Note that the distribution was smooth in color space or in orientation space, not in physical space where colors were randomly presented).

The set size of search displays was either nine (8 distractors + 1 target/distractor) or seventeen (16 distractors + 1 target/distractor) items. The target, if present, was always a conjunction of one extreme color and one extreme orientation (steepest and most blue, steepest and most red, shallowest and most blue, or flattest and most red). The target identity for the entire experiment was specified in advance for each observer. Distractors were always “counter-correlated” with the target. For example, if an observer had to look for a steep and red target, then the distractors followed the following rule: the steeper the line, the bluer it would be. It is easy to see that this rule turns into the standard conjunction search rule (find a red steep line among red flat and blue steep lines) when both colors and orientations are represented by extreme values, that is, when they are both segmentable. On target-absent trials, one randomly chosen distractor replaced the target.

**Figure 2.**
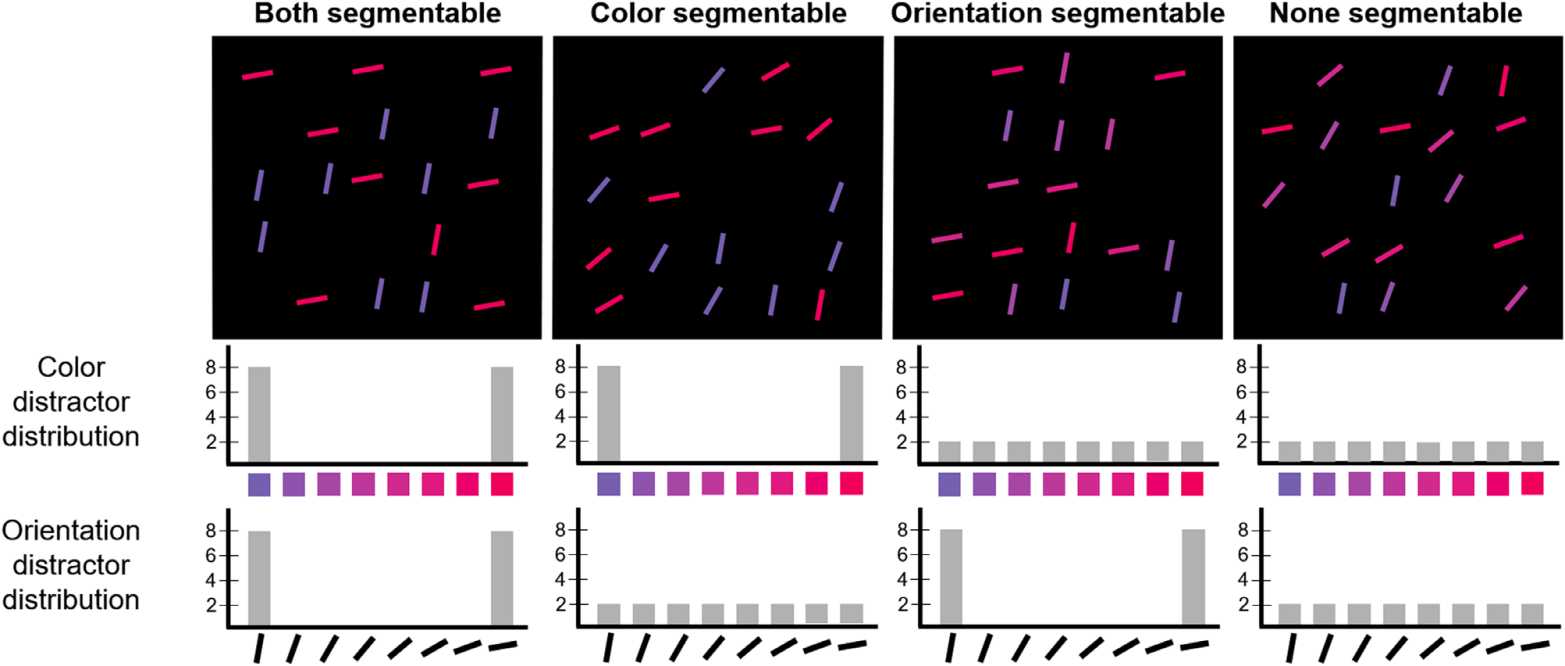
Examples of four segmentability conditions in Experiment 1 for target present trials. Target = *red steep line*, set size = 17. The lines are slightly enlarged for illustrative purposes. The histograms below show frequency distributions of color values and orientation values among distractors.

There were four segmentability conditions: (1) “*both* segmentable” (both orientation and color distractor distribution were sharp, consisting only of extreme colors and orientations), (2) “*none* segmentable” (both orientation and color distribution were smooth, consisting of extremes and transition between them), (3) “*orientation* segmentable” (the orientation distribution was sharp, whereas the color distribution was smooth), (4) “*color* segmentable” (the color distribution was sharp, whereas the orientation distribution was smooth). Figure 2 shows examples of all the types of displays.

#### Procedure

The experiment was run in a darkened room. Participants were seated approximately 50 cm from the screen. They were instructed to look for one of the four possible color-orientation conjunctions. The particular conjunction was consistent for the entire experiment for each participant, with different conjunctions assigned to different participants. The participants had to determine whether the target was present or absent in a display and deliver a speeded response pressing one of two keys assigned for “yes” and “no” responses on a keyboard. Each trial started with the presentation of a fixation point for 500 ms, then a search set was presented until the observer's response or 7000 ms. Responses were followed by feedback (300 ms) informing the observers whether the answer had been correct or not. A next trial started immediately after the feedback.

Each of the four experimental conditions was presented in a separate block of 200 trials preceded by 12 practice trials. The participants could take a break between the blocks. The order of blocks was randomly varied between participants.

### Design and data analysis

In this experiment, we used a 4 (distractor distribution: both, none, orientation, color) × 2 (target: present vs. absent) × 2 (set size: 9 vs. 17) within-subject design. Fifty trials were presented within each cell of this factorial combination, so the total number of trials was 800 per observer. The primary dependent variable was the reaction time (RT) on trials with correct responses. RT changes were then analyzed as a function of set size. The slopes of the RT-set size functions (time per item) were considered to reflect the efficiency of attentional deployment (Wolfe, 1998). The absolute RTs were also analyzed as they could be also informative of processes occurring during search (e.g. stimulus encoding or decision making, which can be relevant to segmentation and categorization studied in this research). Error rates were additionally estimated to control for the speed-accuracy tradeoff. Thus, we did three, repeated-measures analyses of variance (ANOVAs); one each for slopes, average RTs, and error rates with distractor segmentability as the factor of interest in each.

### Results

**Figure 3.**
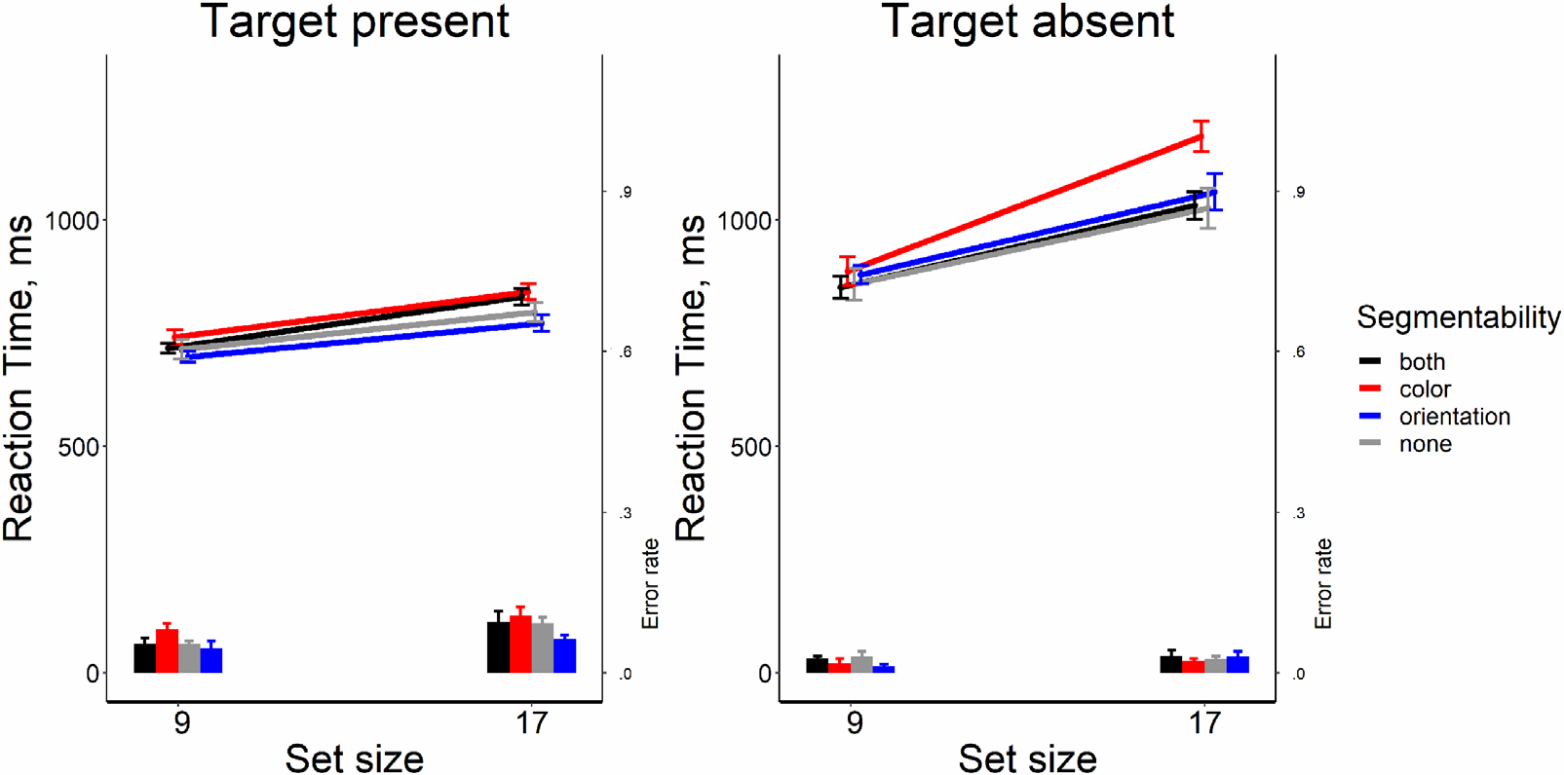
Reaction time and error rate as a function of set size and segmentability in Experiment 1. *Error bars* denote the *SEM,* with between-subject variance removed in accordance with the Cousineau (2005) method.

As shown in Figure 3, it is clear that segmentability did not make search more efficient. The analysis of RT × set size functions slopes showed that all searches were quite efficient (Wolfe, 1998). On the target-present trials, the slopes were between 9 and 14 ms/item, the target-absent trials showed the search rate of 21 to 37 ms/item. Repeated-measure ANOVA for slopes in target-present trials showed no strong evidence for any effect of segmentability as shown by the lack of the main effect distractor condition (*F*[3, 33] = 2.26, *p* = 0.10, η^2^ = 0.17, BF_10_ = 0.95). For target-absent trials, we found some effect of segmentability (*F*[3, 33] = 6.73, *p* = 0.001, η^2^ = 0.38, BF_10_ = 21.92). Post hoc *t*-tests showed that the slopes in the “color segmentable” condition were greater than in other conditions (*t*s[11] > 3.41, *p*s < 0.006, Bonferroni corrected α = 0.008, Cohen's *d*s > 0.98, BF_10_′s > 9.15), whereas the rest conditions did not differ from each other (*t*s[11] < 0.54, *p*s > .60, Bonferroni corrected α = 0.008, Cohen's *d*s < 0.15, BF_10_′s < 0.32). The main results from this experiment are depicted in Figure 3.

The repeated-measure ANOVAs showed the absence of the effect of segmentability on the average RT and on error rates both for target-present and target-absent trials (all *F*s[3, 33] < 1.681 *p*s > .18, η^2^'s < 0.132, BF_10_s < 0.51), except a negligible effect of segmentability for error rates in the target-present condition (*F*[3, 33] = 3.10, *p* = 0.04, η^2^ = 0.22, BF_10_ = 1.71). All pairwise comparisons did not survive multiple-comparison corrections.

## Experiment 2

In Experiment 2, we tested the generality of our conclusions about the effects of segmentability by using a different pair of feature dimensions, length and orientation.

### Participants

In total, 15 undergraduate students at the HSE University (Moscow) participated in Experiment 2 for extra course credits (12 female, mean age 19.25). The results of three participants were excluded from the analysis because they committed more than 20% errors. All participants reported no experience of neurological problems and were tested to show normal or corrected-to-normal visual acuity and no color blindness. At the beginning of experiment, they gave a written informed consent. The protocol complied with the Declaration of Helsinki.

### Apparatus, stimuli, and procedure

**Figure 4.**
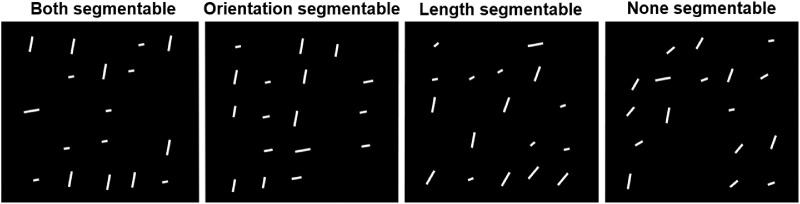
Examples of four segmentability conditions in Experiment 2 for target present trials. Target = *long flat line*, set size = 17. The lines are slightly enlarged for illustrative purposes.

The experiment was developed and presented through PsychoPy for Linux (Peirce et al., 2019). Stimuli were presented on a standard VGA monitor with a refresh frequency of 75 Hz and a 1024 × 768-pixel spatial resolution. Stimuli were similar to Experiment 1 in terms of the spatial arrangement of line elements, their orientation ranges, and how segmentability was defined within each feature dimension. However, as we used length-orientation conjunctions instead of color-orientation conjunctions, there were two important differences: All lines were white and the length of lines varied between 0.45° and 2.46° in steps of 0.29° for the non-segmentable distractors. As in Experiment 1, only the extreme of the length and orientation were used to generate sharp, segmentable length and orientation distributions. As in Experiment 1, we manipulated the segmentability of length and orientation orthogonally (see Figure 4 for examples of all four orthogonal segmentability combinations).

The procedure of the experiment was the same as in Experiment 1. One restriction was made regarding target assignment. Length, unlike color and orientation, is a dimension producing search asymmetries: Search for a long line among short lines is more efficient than vice versa (e.g. Treisman & Gormican, 1988). This asymmetry might cause an additional source of data noise when individual data from observers looking for long or short targets are averaged. To avoid this noise, we assigned our observers only long targets, that is either a long steep line, or a long flat line.

### Design and data analysis

The formal design and data analysis of Experiment 2 were identical with that of Experiment 1.

### Results and discussion

**Figure 5.**
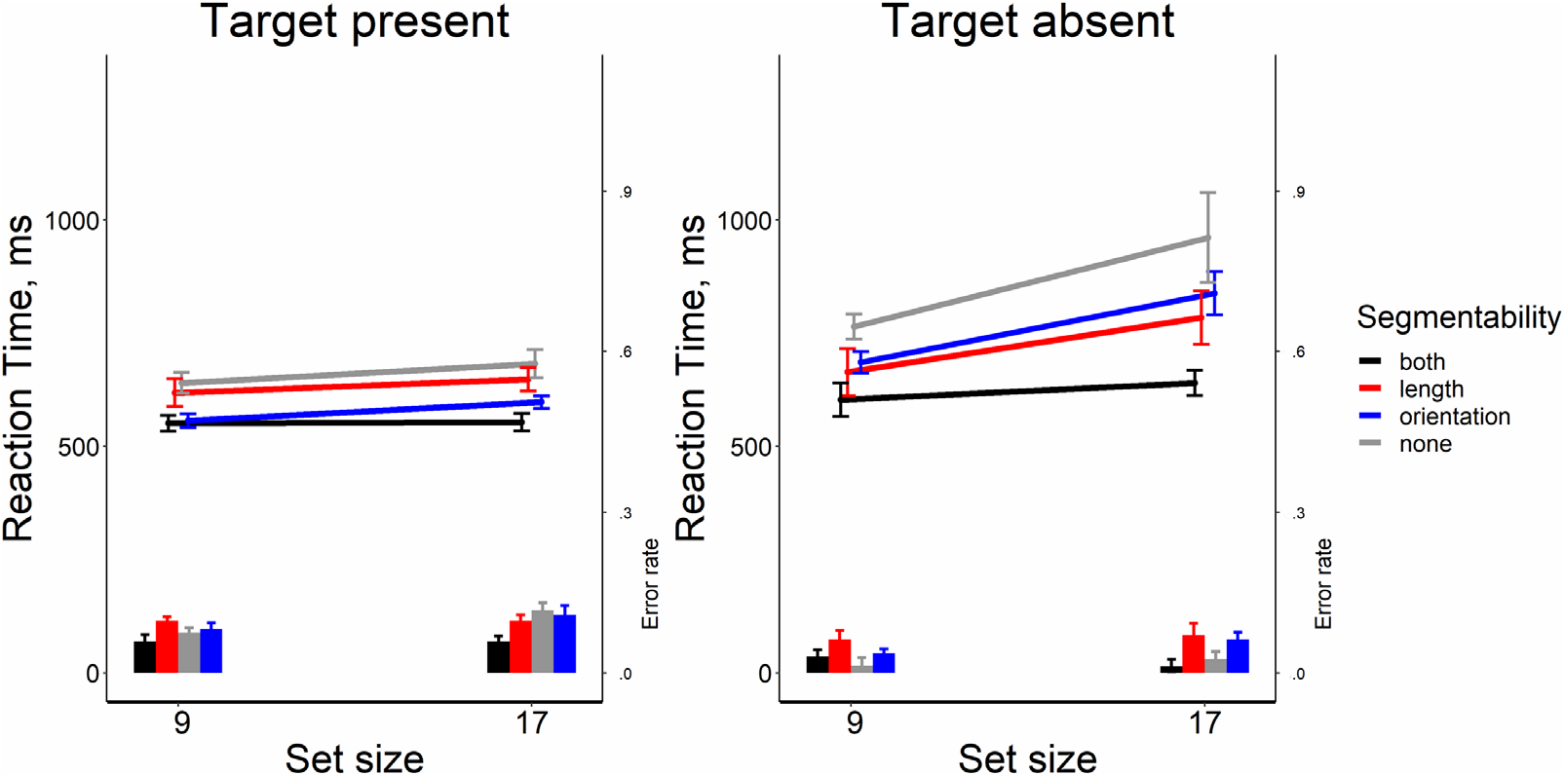
Reaction time and error rate as a function of set size and segmentability in Experiment 2. *Error bars* denote the *SEM,* with between-subject variance removed in accordance with the Cousineau (2005) method.

Figure 5 shows that all of the segmentability conditions of Experiment 2 produce efficient searches. There appears to be a small advantage when both length and orientation are segmentable, but it is quite weak statistically. When both length and orientation were segmentable, the slopes were 0 ms/item in the target-present and 5 ms/item in the target-absent trials suggesting an extremely efficient conjunction search comparable with the pop-out feature search. In the other three segmentability conditions, the slopes were slightly greater but again showed very efficient target present search slopes (4-5 ms/item in the target absent and 15 to 24 ms/item in the target-absent trials). In line with the finding of efficient search in all conditions, we found no effect of distractor segmentability on slopes in target-present trials (*F*[3, 33] = 1.73, *p* = 0.18, η^2^ = 0.14, BF_10_ = 0.62). The effect for target-absent trials is significant (*F*[3, 33] = 3.51, *p* = 0.03, η^2^ = 0.24, BF_10_ = 2.6), although none of the pairwise comparisons of target-absent conditions was below the significance threshold corrected for multiple comparisons (*t*s[11] < 2.98, *p*s > 0.012, Bonferroni corrected α = 0.008, Cohen's *d*s < 0.86, BF_10_s < 4.90).

There is a small effect of segmentability on the average RTs both in target-present (*F*[3, 33] = 4.11, *p* < 0.14, η^2^ = 0.27, BF_10_ = 3.89) and in target-absent trials (*F*[3, 33] = 3.51, *p* < 0.026, η^2^ = 0.24, BF_10_ = 2.44). Again, none of the comparisons between conditions survived correction for multiple comparisons (Bonferroni corrected α = 0.008). Some evidence was found for the effect of segmentability on error rates in the target-present condition (*F*[3, 33] = 4.97, *p* = 0.006, η^2^ = 0.31, BF_10_ = 7.63) mostly provided by a slightly smaller error in the “both segmentable” displays.

The results of Experiment 2 are similar with the results of Experiment 1 that used exactly the same design but a different pair of features. While there seems to be a small advantage for searches where both features were segmentable, that effect is statistically quite weak. More importantly, all conditions produced efficient searches making it clear that the lack of clear groups of orientations or lengths did not prevent guidance by those features in a conjunction task.

### Interim discussion

The main finding in Experiments 1 and 2 was that the typical efficiency of color × orientation and length × orientation conjunction searches was basically not affected by the segmentability manipulations. This is especially clear for target-present displays. We can conclude from these results that grouping is not critical for the efficiency of conjunction search. The lack of the segmentability effect might seem to be an unusual result, given the previous robust evidence for the role of grouping, segmentation, heterogeneity, and so on in visual search (Avraham, Yeshurun & Lindenbaum, 2008; Duncan & Humphreys, 1989; Palmer, Verghese & Pavel, 2000; Rosenholtz, 2001; Rosenholtz, Huang & Ehinger, 2012; Wolfe et al., 1992). Moreover, we found substantial effect of segmentability using stimuli very similar to those used in Experiment 2 but in a different texture discrimination task (Utochkin, Khvostov & Stakina, 2018). Therefore the lack of an effect of segmentability cannot be attributed to a flaw in the stimuli, since those stimuli can produce convincing effects. Indeed, as will be seen in Experiments 3 and 4, segmentability can have a greater effect on search performance with these stimuli when specific requirements of the conjunction search task are changed or when feature search is performed.

**Figure 6.**
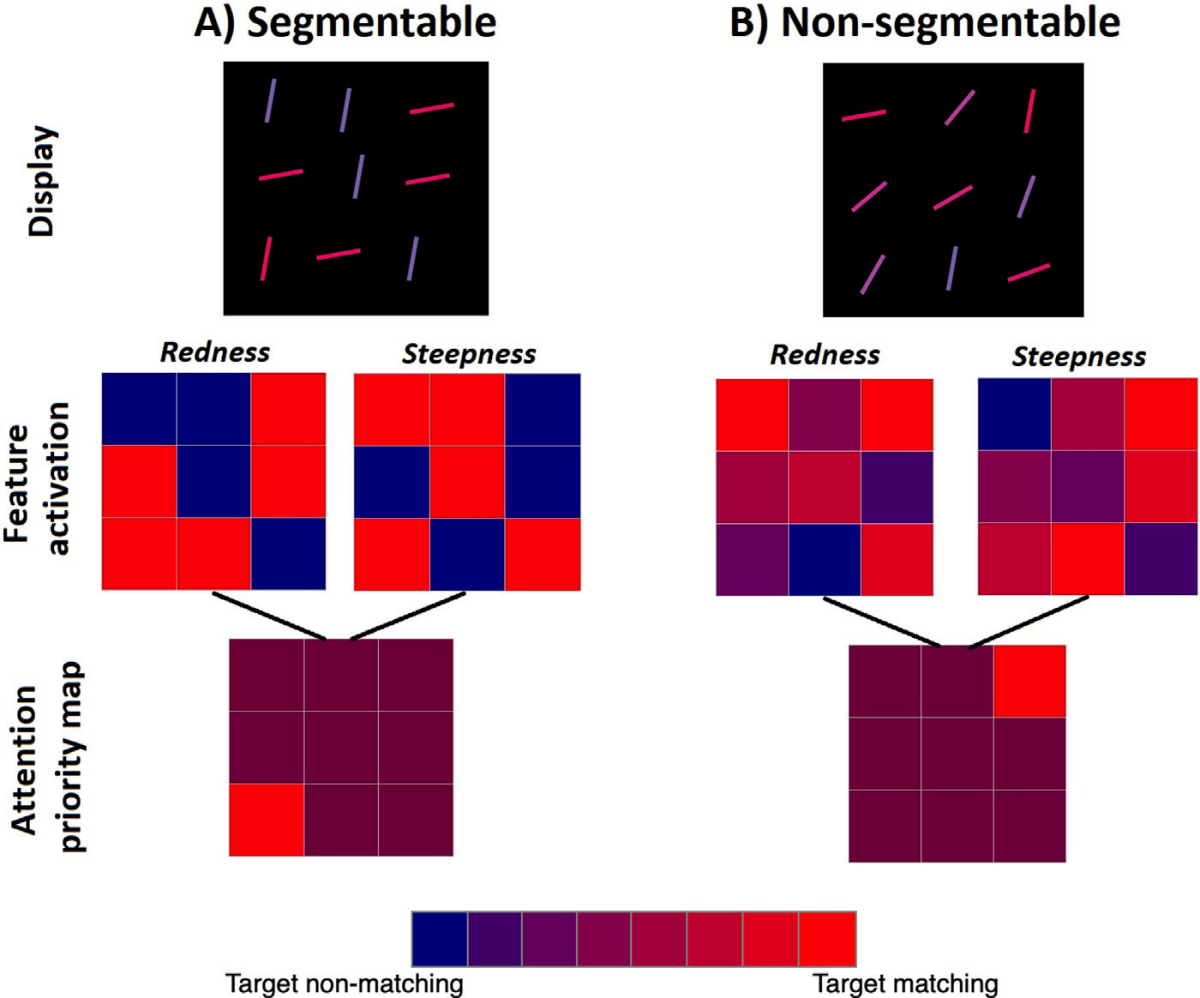
A Guided search (Wolfe, 1994; Wolfe et al., 1989) account for the absence of the effect of segmentability on conjunction search in Experiments 1 and 2. The proposed mechanism is based on the parallel use of top-down activation patterns from color (“redness”) and orientation (“steepness”) feature map. In (A), highly segmentable colors and orientations both provide distinct patterns of strongly activated and weakly activated locations, and their overlap leads to an attentional priority map with many moderately activated locations and one highly activated location corresponding to the target. In (B), non-segmentable colors and orientations both provide fuzzy patterns of top-down activation, but their overlap looks similar to the pattern in (A). Colors on Feature activation maps represent hypothetical top-down activation weighted by the similarity of a feature at a given location with the target feature. Each location on the Attention priority map is colored as the average of activation values in corresponding locations of the two Feature activation maps.

Another, potentially more plausible account of our main result can be based on the Guided Search model (Wolfe, 1994; Wolfe et al., 1992). Guided Search would explain the efficiency of color × orientation or length × orientation searches by proposing that attention would be guided simultaneously toward red and toward steep in a search for a red steep item. Though all the items in a standard conjunction search would be *either* red or steep, only a target item would be both red *and* steep. Its double dose of guidance would attract attention to the target location. As shown in Figure 6, the situation is not much different for the non-segmentable conditions. The target gets a double dose of guidance, distractors get less because as one guiding feature (e.g. redness) gets stronger, the other (steepness) gets weaker (due to opposite directions of correlation between color and orientation for target and distractors). Importantly, according to Guided Search model (Wolfe, 1994; Wolfe et al., 1989), simultaneous guidance means that during conjunction search, attention operates with the information from the Attention priority map. Although this map uses different Feature activation maps of all locations as inputs (see description of Figure 6 for details), the search is ultimately guided by the output of the priority map and can make use of both sources of guidance at the same time.

Actually, looking at Figure 6, it would seem that the target should always have the highest activation on the Attentional priority map. If this were true, these conjunction searches should be like feature searches. Attention should be summoned to the target first on each target-present trial and the slopes of RT x Set Size functions should be near zero. This problem has been discussed in various descriptions of Guided Search (Wolfe, 1994). In brief, the proposed answer is that the guidance is corrupted by various sources of noise. The result is that attention is biased toward the target, but imperfectly. As a consequence, conjunction searches of this sort are more efficient than unguided searches, for example, for a T among Ls, but less efficient than search for a red done among blue or a steep line among shallow lines.

This model makes an interesting distinction between feature and conjunction search. Although the outputs on the attention priority maps are very similar between Figures 6A and 6B, the patterns of activation on the component feature maps are clearly different. The patterns are closer to all-or-none for the segmentable feature distributions (Figure 6A) and more gradual, fuzzy for the non-segmentable distributions (Figure 6B). This predicts that the effects of segmentability would be revealed if simultaneous guidance by two features is limited. This prediction is tested in Experiments 3 and 4. When the task is structured so that simultaneous guidance by both features is precluded, then we can see the benefits of segmentation and grouping that are not seen in Experiments 1 and 2.

The model of guidance suggested in Figure 6 implies that the superiority of top-down guidance by two features over grouping and segmentation comes from an ability to use the features simultaneously. That is, that top-down activations from both feature maps are summed at all locations without any prior subsetting and attention is guided to a location with the highest total activation (Wolfe, 1994). An opposing view is that each of the features gets used strictly in turn. For example, during the search for a red vertical item, attention might be guided first to a RED subset and then to a VERTICAL item in that subset, according to this sequential account (e.g. Huang & Pashler, 2007). A sequential theory would seem to have some difficulty in explaining the results of Experiments 1 and 2, because it would predict at least some effect of segmentability. If the display is not segmentable, it should be harder to group items into useful subsets (e.g., group all red items and then look for a singleton in that subset), because there are a lot of items producing at least some fuzzy target signal (Figure 6B). Moreover, non-segmentable displays also increase distractor heterogeneity. As a result, target segmentation within the selected subset should also be harder, which would make subset search even slower (e.g., finding the steepest line among many different tilts can be harder than among flattest line, although it is a matter of target-distractor difference, Foster & Ward, 1991). Yet, at this point in our study, we cannot totally rule out the possibility of sequential guidance based on the absence of the segmentability effect in Experiments 1 and 2. With some additional assumptions, the sequential theories can account for the current results. For example, if we assume that participants could guide their attention to the reddest items, they would create some coarse subset. A non-segmentable “subset” would not be homogeneous or perceptually obvious, but participants still could perform orientation search over the subset and do the task. Alternatively, participants might be able to create feature subsets with exceptional precision (e.g., guiding attention only to extremely red items) in which case the distribution of other features would not matter. However, by the time observers making a subset of just one or two red items, this sounds a lot like standard attentional guidance to the red item. To address these and related ideas, Experiment 4 and, in particular, Experiment 3 further examine the question which of these mechanisms—sequential or simultaneous—is more likely involved in performing the conjunction search task in our case.

## Experiment 3

In Experiment 3,  we tested whether the segmentability of distractors affects conjunction search if top-down guidance is limited to one feature. We accomplished this using a “subset search” paradigm, a modification of the standard conjunction search task (Friedman-Hill & Wolfe, 1995). Participants looked for a conjunction target with one predefined, known feature (color) and another unknown feature (here, orientation) that can only be identified once the color subset has been found. For example, participants could be asked to look for a line with a unique orientation in a subset of red (or reddish) lines. In contrast to the standard conjunction search, subset search forces a sequential mode of guidance, since search for an odd element in orientation can start only after a relevant color subset is selected (although these two stages do not need to be entirely sequential, Friedman-Hill & Wolfe, 1995). As in Experiment 1, we tested search efficiency when both colors and orientations were segmentable, both were non-segmentable, and either color or orientation, alone, was segmentable. The most important difference from our core experiment (Experiment 1) was that color-orientation combinations for distractors randomly changed from trial to trial. In some trials, bluish distractors were steeper and reddish distractors were flatter; in other trials, reddish distractors were steeper and bluish distractors were flatter (targets had the opposite pattern).

This experiment is designed to encourage sequential guidance by inducing observers to select a subset based on one feature and then to perform a feature search based on the other feature. This limits an immediate access to top-down information about one of two relevant features. It is reasonable to expect that segmentability would encourage the formation of that first subset and would make the search easier, thus revealing the contribution of grouping and segmentation at the level of separate feature maps (see the model suggested in Figure 6). If observers somehow use simultaneous guidance, the search efficiency should not differ. In this case, we expect that only the average RT would increase in non-segmentable conditions because it would be more difficult to determine a target orientation if one or both features are non-segmentable. This experiment should show us the pattern of data produced by sequential strategy of search (or at least partially sequential because participants always need to select the relevant color subset at first). From those results, we can better determine if the patterns of results in the earlier experiments look like they could have come from sequential guidance.

### Method

#### Participants

In total, 13 students at the HSE University participated in Experiment 3 for extra course credits (11 female, mean age 20.33). The results of one participant were excluded from the analysis because of an error rate of more than 20%. All participants reported no experience of neurological problems and showed normal or corrected-to-normal visual acuity and no color blindness. At the beginning of the experiment, they gave a written informed consent.

#### Apparatus, stimuli, and procedure

**Figure 7.**
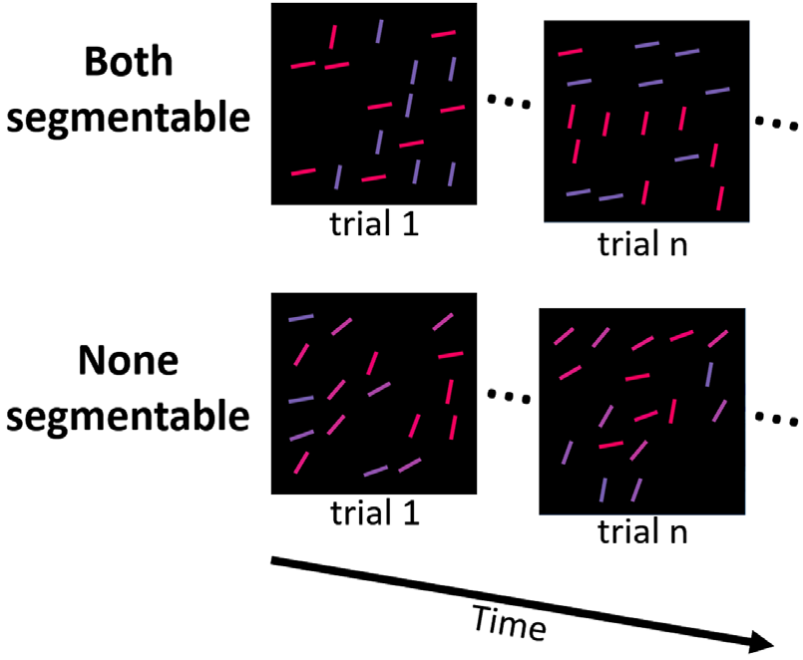
Examples of the subset search task for the “both segmentable” (*top row*) and “none segmentable” conditions. In each trial, observers have to look for an odd orientation among *red* (or *reddish*) *lines*. In each trial shown as a separate array on the figure, the direction of correlation between color and orientation could randomly change that led to unpredictable changes in orientations between targets and distractors within the target color subset. The *lines* are slightly enlarged for illustrative purposes.

We used the same apparatus as in Experiment 2. Stimuli were identical to Experiment 1 in terms of the spatial arrangement of line elements, their color and orientation ranges, and how segmentability was defined within each feature dimension. However, there was an important change regarding target assignment. Participants were always trying to find an orientation outlier in a colored subset. For each participant, only a target color was consistently defined. For one half of participants, the target color was red, for the other half of participants, it was blue. As for the target orientation, it was variable across trials, which entailed an unpredictable change in both the target conjunction and the distractor conjunction rule. For example, suppose an observer was instructed to look for a singleton orientation in the blue (or bluish) subset. Then, on some trials, the target would be a steep blue line among distractors with a conjunction rule “the steeper the line, the redder it is.” In other trials, the target will be a shallow blue line among distractors with a conjunction rule “the flatter the line, the redder it is” (Figure 7).

As in Experiment 1, we orthogonally manipulated the segmentability of colors and orientations, which yielded four blocks of trials. In addition, we have run a block with a standard conjunction search task (both the color and orientation of a target are consistent) with both features having the segmentable distributions. We used this block as a standard baseline to compare subset search performance with.

#### Design and data analysis

In this experiment, we had a 5 (segmentability: both, none, orientation, color + one standard conjunction search) × 2 (target: present vs. absent) × 2 (set size: 9 vs. 17) within-subject design. 50 trials were presented within each factorial combination, so the total number of trials was 1000 per observer. The dependent variables and their analyses were the same as in Experiment 1.

### Results and discussion

**Figure 8.**
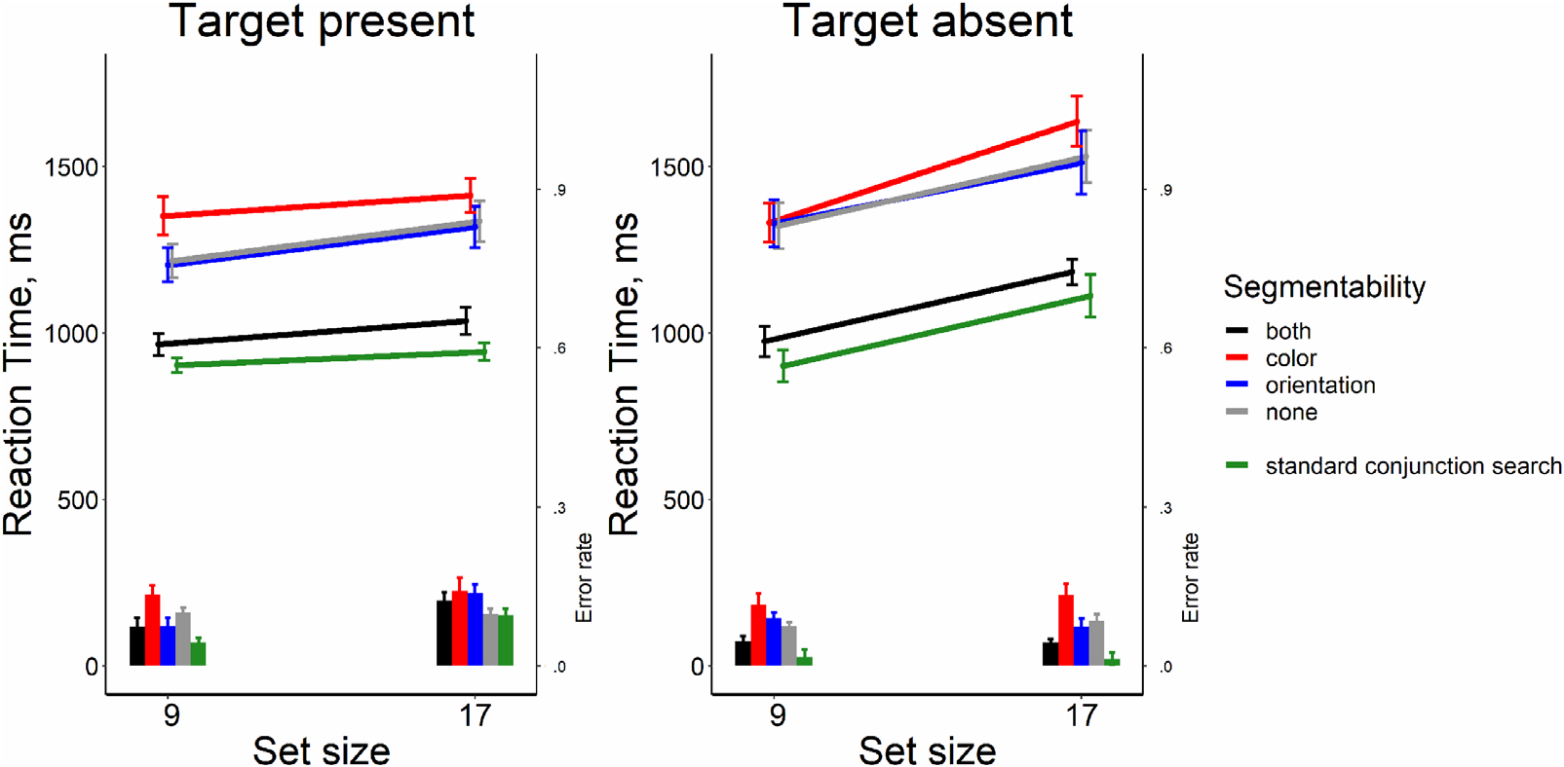
Reaction time and error rate as a function of set size and segmentability in Experiment 3. *Error bars* denote the *SEM,* with between-subject variance removed in accordance with the Cousineau (2005) method.

RT x set size functions are shown in Figure 8. It should be clear that all the subset searches are slower than the standard conjunction search. This is particularly true of the conditions in which one or both of the features were non-segmentable (target present trials: *F*[4, 44] = 14.99, *p* < 0.001, η^2^ = 0.58, BF_10_ = 1.52 × 10^5^; target absent trials: (*F*[4, 44] = 9.77, *p* < 0.001, η^2^ = 0.47, BF_10_ = 2.13 × 10^3^). Looking in more detail, the standard conjunction search and the fully segmentable subset search were faster than any subset search in which either or both feature distributions were non-segmentable (target present: (*t*s[11] > 3.48, *p*s < 0.005, Bonferroni corrected α = 0.005, Cohen's *d*s > 1.01, BF_10_s > 10.23; target absent: *t*s[11] > 3.57, *p*s < 0.004, Bonferroni corrected α = 0.005, Cohen's *d*s > 1.03, BF_10_s > 11.61, except for the difference between the “both segmentable” and “orientation segmentable” in target-absent trials that turned out to be greater than the Bonferroni correction, *p* = 0.009).

In contrast with the absolute speed of search, search efficiency was not strongly affected by the task and segmentability (target-present trials: *F*[4, 44] = 1.56, *p* = 0.20, η^2^ = 0.12, BF_10_ = 0.55); target-absent: *F*[4, 44] = 2.16, *p* = 0.09, η^2^ = 0.16, BF_10_ = 0.95). Overall, our observers showed quite efficient searches in all of the subset search tasks (8-14 ms/item in target-present and 22-26 ms/item in target absent trials, except for the “color segmentable” trials with 38 ms/item in the target-absent condition). The reference task, standard conjunction search also provided comparable search rates (5 ms/item for target-present and 26 ms/item for target-absent).

We also found modest effects of segmentability on error rates. For target-present trials, the overall ANOVA model found a slight effect (*F*[4, 44] = 3.82, *p* = 0.01, η^2^ = 0.258, BF_10_ = 5.16), though pairwise comparisons did not exceed the significance threshold, once corrected for multiple comparisons (*t*s[11] < 3.076, *p*s > .01, Bonferroni corrected α = 0.005, Cohen's *d*s < 0.89, BF_10_′s < 5.68). For target absent trials, evidence for the effect of segmentability on the error rate (false alarms) was stronger (*F*[4, 44] = 8.67, *p* < 0.001, η^2^ = 0.44, BF_10_ = 826). The false alarm rate in the standard conjunction search (2%) was smaller than in the “none segmentable” and “orientation segmentable” subset searches (8% in each; *t*[11] > 4.29, *p* < 0.002, Bonferroni corrected α = 0.005, Cohen's *d* > 1.24, BF_10_ > 32.62), whereas the rest of the conditions did not differ from each other (*t*s[11] < 3.41, *p*s > 0.005, Bonferroni corrected α = 0.005, Cohen's *d*s < 0.99, BF_10_′s < 9.23).

Overall, the results of Experiment 3 suggest that subset search had almost the same efficiency as the conjunction search in terms of slopes of the RT × set size functions. This resemblance of search slopes between conjunction and subset searches was previously demonstrated by Friedman-Hill and Wolfe (1995) when they used segmentable feature distributions. Our Experiment 3 adds the new finding that non-segmentable subset searches remain efficient. It is absolute RTs that turn out to be sensitive to segmentability. Overall, RTs were ∼200-300 ms higher when one or both dimensions was non-segmentable (Figure 8). One plausible explanation for this pattern is that reducing segmentability makes it more difficult for observers to determine what the target orientation will be for that trial. Once the participant figures out whether this target must be steep or shallow, the task becomes a typical conjunction search, where observers can deploy attention in a similar manner in segmentable and non-segmentable conditions. Hence, we observed no change in the slopes of the RT x set size functions, but the significant effect of segmentability on the average RTs. Therefore, Experiment 3 provides an interesting dissociation between processes underlying global segmentation and categorization and the guided deployment of attention. Even when the role of preliminary categorization is artificially increased, it turns out that search itself is likely guided by two features at one time. Thus, it could be that segmentability was only useful in defining the target, not in conducting the search.

## Experiment 4

In Experiments 1 and 2, we demonstrated that the effect of segmentability on the efficiency of conjunction search was small or non-existent. Our model (Figure 6) suggests, the superposition of two feature activation maps leads to similar output patterns on the Attention priority map, although the feature maps show substantially different patterns between the segmentable and non-segmentable cases. This, in turn, implies that, even if we do not see the effects of segmentability on conjunction search, we could see effects on feature search, using exactly the same stimuli. Normally, we would expect feature searches to be more efficient than search for a conjunction of those features. In standard versions of the tasks, search for “red” among blue or “steep” among flat would be more efficient than search for a “red steep” among blue steep and red flat. However, this might not be the case with non-segmentable feature distributions, using stimuli like those in the previous experiments. In Experiment 4, we replicated the segmentable and non-segmentable conjunction searches from Experiment 1 using somewhat different stimuli. In addition, for the critical comparison, we tested the color and orientation feature searches with the non-segmentable stimuli. We did not feel the need to replicate the segmentable feature searches since there is no doubt that search for a salient color or orientation among homogeneous distractors will be highly efficient searches with slopes near zero.

### Method

#### Participants

In total, 14 undergraduate students at the HSE University (Moscow) participated in Experiment 4 for extra course credits (11 female, mean age – 19.8). The results of one participant were excluded from the analysis because of an error rate of more than 20%. All participants reported no experience of neurological problems and were shown to have normal or corrected-to-normal visual acuity and no color blindness. At the beginning of experiment, they gave a written informed consent.

#### Apparatus, stimuli and procedure

We used the same apparatus as in Experiment 2. Our stimuli were similar to Experiment 1 in terms of line sizes, set sizes, and spatial arrangement. As before, we manipulated the distributions of colors and orientations, although these distributions were created in a different way than in Experiment 1. The orientation of lines varied between strictly vertical (0°) and strictly horizontal (90°) in seven steps of 12-13°. The orientation steps could be clockwise (0°, 13°, 26°, …, 90°) or counter-clockwise (0°, –13°, –26°, … −90°). This produced two symmetrical orientation distributions (“left” and “right”). Left and right distributions were randomly assigned to different trials in conditions where orientation was non-segmentable. For segmentable orientation distributions, clockwise or counterclockwise rotation did not have any meaning since the distributions consisted only of vertical and horizontal lines. The color manipulation involved the variation in the saturation of the red color within the HSV (hue-saturation-value) space. The range of saturation varied from 0 (completely white) to 1 (saturated red) with steps of 1/7, providing transition through various shades of red and pink. The extreme values of color (red, white) and orientation (vertical, horizontal) were used to create the sharp, segmentable distributions. Non-segmentable, smooth distributions were created by presenting all the steps of orientations and colors. These changes in the color and orientation distributions (compared to Experiment 1) aimed to diminish potential confusability between the target and similar non-target features that we anticipated in our feature search tasks. Intuitively, a strictly vertical line among 10°-tilted distractors seems to be a better-defined target than a 10°-tilted line among 20°-tilted lines. Similarly, a totally white line among slightly pinkish lines can be a more recognizable target than a reddish line amongst slightly bluer lines as in Experiment 1. Following the design of Experiment 1, our participants performed a conjunction search task with segmentable and non-segmentable distractors. The conjunction target was defined as either white vertical, or white horizontal line. In this experiment, we used only the fully segmentable and fully non-segmentable conjunction search conditions.

**Figure 9.**
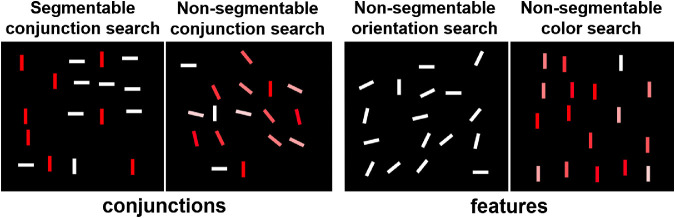
Examples of four search tasks in Experiment 4 for target present trials. Target = *white vertical line* (only white for color search, only vertical for orientation search), set size = 17. The lines are slightly enlarged for illustrative purposes.

In addition to the conjunction search, our participants also performed two feature search tasks, one for color and one for orientation. In the color search task, all items had the same orientation (horizontal or vertical) and differed only in color (saturation). The target, if present, was a completely desaturated (“white”) line, whereas distractors could have all of the rest saturation levels, including the maximum (“red”). In the orientation search task, all lines were white and differed only in orientation. The target could be either horizontal, or vertical (consistent within observer but varying between observers). The distractors included all the other orientations in the non-segmentable set. Figure 9 shows example stimuli for all four tasks. As noted, we did not test features searches with the segmentable distractors since we know that a white target among saturated-red distractors or a vertical target among horizontal distractors will produce essentially flat RT x set size functions.

Each task was presented in a separate block of 200 trials preceded by 12 practice trials with a rest break between the blocks. The serial order of the blocks was randomized across participants.

#### Design and data analysis

In this experiment, we used a 4 (search task: conjunction search in segmentable displays, conjunction search in non-segmentable displays, color search, orientation search) × 2 (target: present vs. absent) × 2 (set size: 9 vs. 17) within-subject design. The dependent variables and their analyses were the same as in Experiment 1.

### Results and discussion

**Figure 10.**
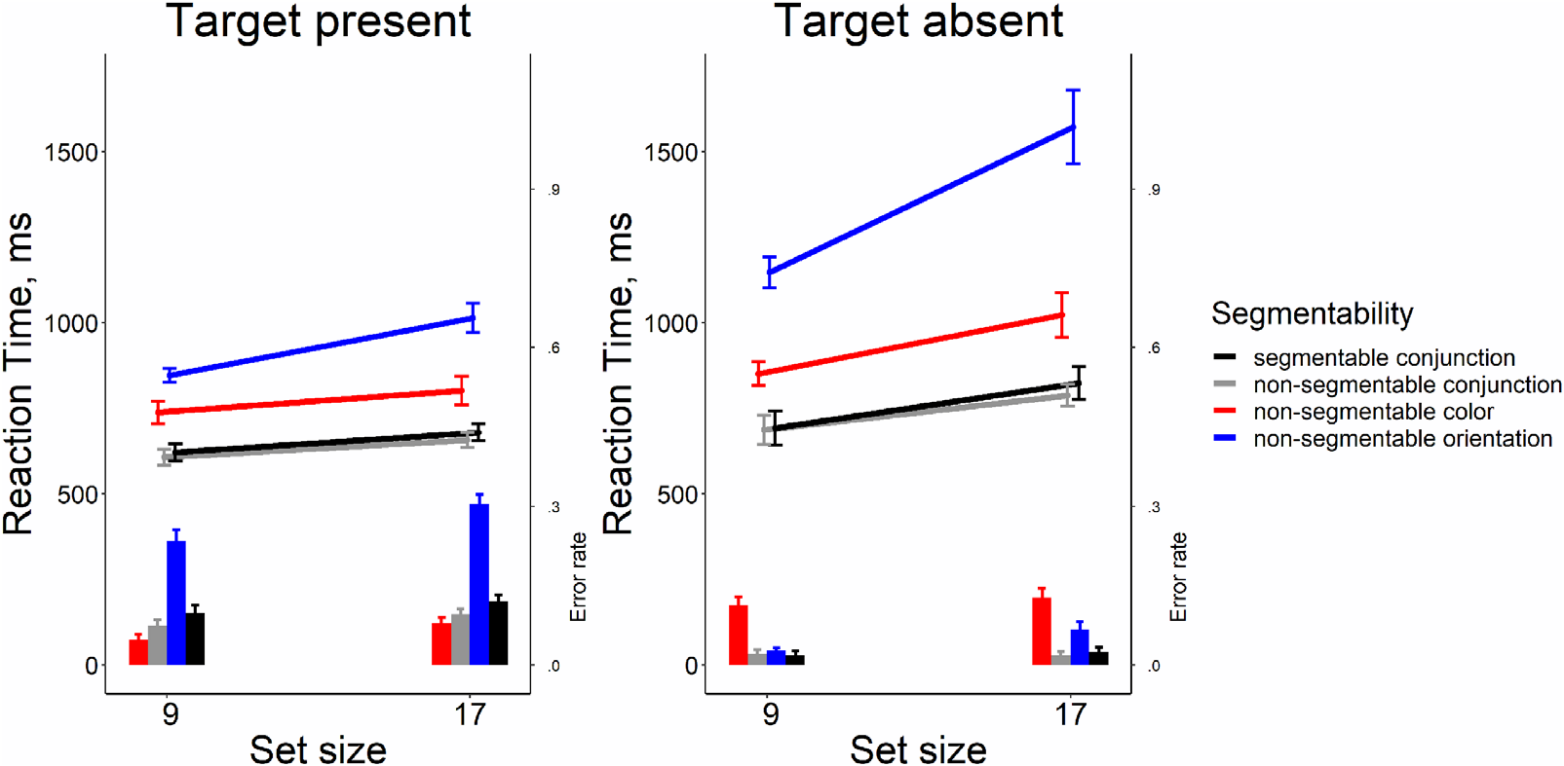
Reaction time and error rate as a function of set size and task in Experiment 4. Error bars denote the *SEM,* with between-subject variance removed in accordance with the Cousineau (2005) method.

Figure 10 reveals an unusual pattern of results. The conjunction search is easier (or, at the very least, certainly not harder) than the component feature searches. As in Experiment 1, we found that slopes of the RT × set size functions were quite efficient for segmentable and non-segmentable conjunction search tasks (6-7 ms/item in target-present and 13-17 ms/item in the target-absent trials). For color feature search, the slopes were also reasonably efficient search (8 ms/item in the target-present and 21 ms/item in the target-absent trials). For orientation feature search, the slopes were inefficient (21 ms/item in the target-present and 53 ms/item in the target-absent trials.

For target-present trials, we found the strong effect of the search task on the slope (*F*[3, 39] = 12.74, *p* < 0.001, η^2^ = 0.50, BF_10_ = 1.07 × 10^4^). Pairwise comparisons showed that slopes in the orientation search task were much steeper than in other conditions (*t*s[13] > 3.57, *p*s ≤ 0.003, Bonferroni corrected α = 0.008, Cohen's *d*s > 0.95, BF_10_s > 13.79, Figure 10). The color feature search did not differ from the conjunction tasks (*t*s[13] < 0.8, *p*s > .438, Bonferroni corrected α = 0.008, Cohen's *d*s < 0.215, BF_10_′s < 0.357). For target-absent trials, the pattern was the same (*F* (3, 39) = 15.98, *p* < 0.001, η^2^ = 0.55, BF_10_ = 6.85×10^4^), with the orientation search task producing much greater slopes than other three tasks (*t*s[13] > 4.10, *p*s < 0.0011, Bonferroni corrected α = 0.008, Cohen's *d*s > 1.1, BF_10_′s > 32.53, Figure 10) with the rest of conditions not differing from each other (*t*s[13] < 2.07, *p*s > .06, Bonferroni corrected α = 0.008, Cohen's *d*s < 0.55, BF_10_′s < 1.37).

We also found similar effects of the task on the average RTs in target-present trials (*F*[3, 39] = 16.77, *p* < 0.001, η^2^ = 0.56, BF_10_ = 7.92 × 10^4^). Again, the main effect was driven by the orientation search task yielding a greater average RT than the two conjunction searches (*t*s[13] > 6.38, *p*s < 0.001, Bonferroni corrected α = 0 .008, Cohen's *d*s > 1.70, BF_10_s > 1.01 × 10^3^), whereas other conditions did not differ from each other (*t*s[13] < 2.97, *p*s > .01, Bonferroni corrected α = 0.008, Cohen's *d*s < 0.79, BF_10_′s < 5.23). For target-absent trials, the main effect was also present (*F*[3, 39] = 22.08, *p* = 0.001, η^2^ = 0.63, BF_10_ = 1.82 × 10^6^). The orientation search average RT was bigger than the RTs in the rest of the conditions (*t*s[13] = 4.50, *p*s < 0.001, Bonferroni corrected α = 0.008, Cohen's *d*s = 1.20, BF_10_s = 195.43), while the rest of the tasks did not differ (*t*s[13] < 2.96, *p*s > 0.01, Bonferroni corrected α = 0.008, Cohen's *d*s < 0.79, BF_10_s < 5.23).

Finally, we found that the different tasks produced different error rates in both target-present (*F*[3, 39] = 58.6, *p* < 0.001, η^2^ = 0.82, BF_10_ = 1.48 × 10^13^) and target-absent (*F*[3, 39] = 16.71, *p* < 0.001, η^2^ = 0.56, BF_10_ = 2.3 × 10^5^) trials. In target-present trials, this effect was basically provided by the orientation search with an average of 27% errors (“miss” responses) that was greater than in the rest of the tasks with an average of 6% to 11% misses (*t*s[13] > 6.65, *p*s < 0.001, Bonferroni corrected α = 0.008, Cohen's *d*s > 1.78, BF_10_s > 1478). We also found that segmentable conjunction search yielded a slightly bigger rate of miss errors than the color search (*t*[13] = 3.71, *p* = 0.004, Bonferroni corrected α = 0.008, Cohen's *d* = 0.99, BF_10_ = 17.259). The rest of the conditions did not differ from each other in terms of the miss errors (*t*s[13] < 2.62, *p*s > 0.02, Bonferroni corrected α = 0.008, Cohen's *d*s < 0.7, BF_10_s < 3.08). For target-absent trials, the pattern of errors (“false” alarms) was different. Here, we found that the color search task produced ∼12% false alarms on average; a rate that was higher than in the other tasks (∼2%–5% false alarms, *t*[13] > 3.62, *p* < 0.004, Bonferroni corrected α = 0.008, Cohen's *d* > 0.97, BF_10_ > 14.87), whereas the remaining conditions did not differ from each other (*t*s[13] < 2.54, *p*s > 0.02, Bonferroni corrected α = 0.008, Cohen's *d*s < 0.68, BF_10_s < 2.75).

In Experiment 4, we replicated the finding from Experiment 1 that conjunction search can be quite efficient and is not affected by the segmentability of distractors. The novel aspect of the Experiment 4 results is the finding that the non-segmentable feature searches were harder than the conjunction searches. In the case of the orientation search, the feature searches were markedly less efficient. The feature search is more sensitive to non-segmentability than conjunction search because conjunction search can make better use of feature guidance than the feature searches in this case. The combination of two relatively noisy feature activation maps produces a combined priority map that can do quite a good job of guiding attention to a conjunction target (as suggested by the model in Figure 6). Therefore our results provide evidence that feature grouping and segmentation, as well as heterogeneity, in general, are important determinants of search efficiency, but top-down guidance can be powerful enough to overcome their effects on search. The detrimental effect of poor segmentability, observed in this experiment, may seem opposite to the facilitating effect found in Utochkin and Yurevich (2016). However, both effects are flip sides of the same mechanism. In Utochkin and Yurevich (2016), the target was always highly distinct from all distractors (see the example in Figure 1 of the present article), so non-segmentable distractors provided stronger background grouping and better target segmentation. In the present experiment, the target feature was always a part of the non-segmentable group, so it was harder to detect it.

**Figure 11.**
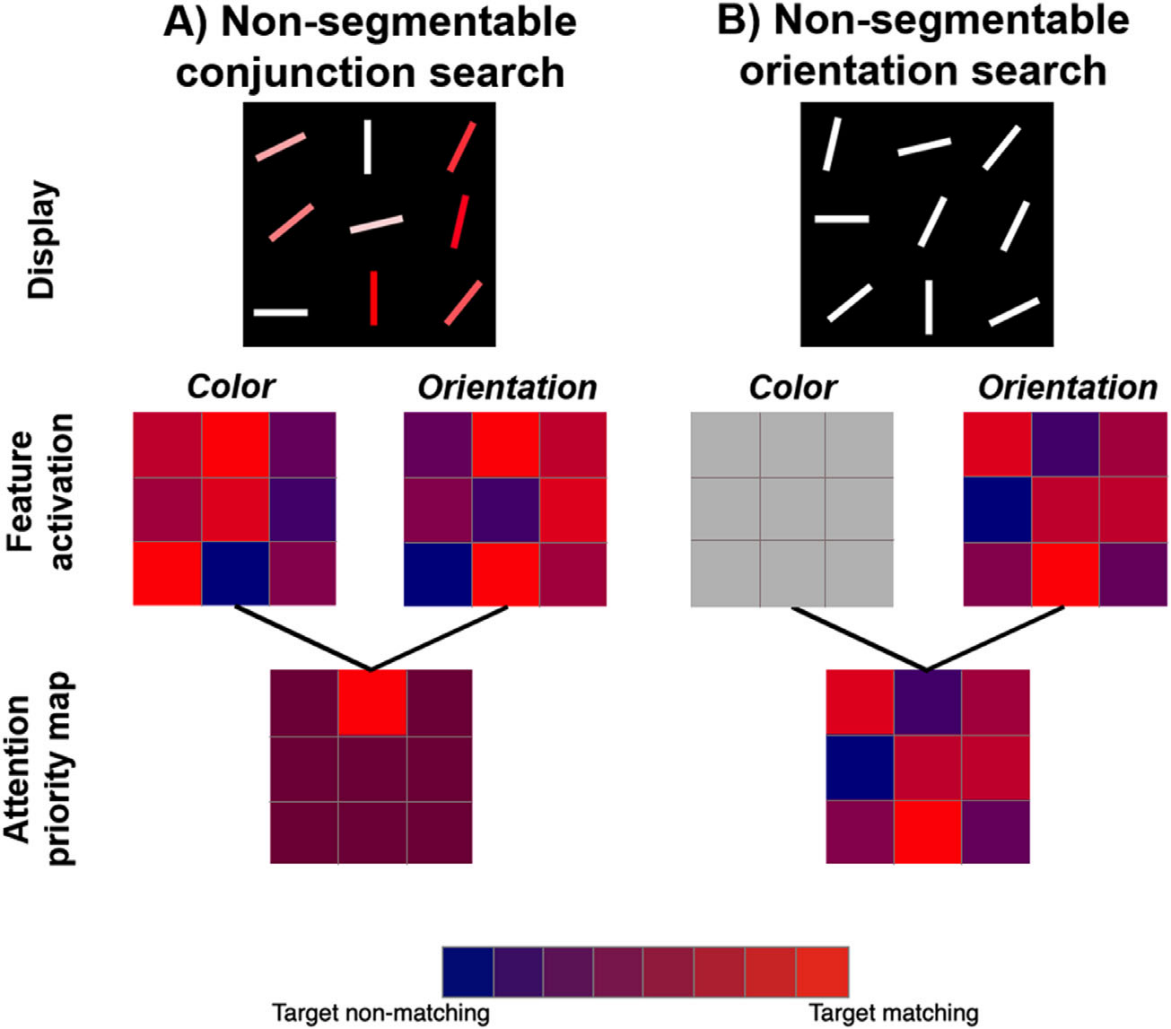
A Guided search (Wolfe, 1994; Wolfe et al., 1989) account of the results of Experiment 4. The proposed mechanism is based on the simultaneous use of top-down activation patterns from color (“redness”) and orientation (“steepness”) feature map. The color scheme for the Feature activation maps and Attention priority map is the same as in Figure 6. In (A), a combination of non-segmentable colors and orientations both provide fuzzy patterns of top-down activation, but their overlap produces an attentional priority map with many moderately activated locations and one highly activated location corresponding to a target. As a result, the search is quite efficient. In (B), non-segmentable orientations provide a fuzzy pattern of top-down activation, whereas color distribution with only one value provides no relevant information (thus shown in gray); therefore their overlap looks as fuzzy as the orientation map alone. As a result, the search becomes less efficient.

At first glance, the fact that feature search is harder than conjunction search is at odds with one of the standard findings of visual search literature (Treisman & Gelade, 1980). However, if one accepts that conjunction search can be guided by several features simultaneously, this result is showing that search becomes easier as the difference between the target and distractors increases. As is obvious from Figure 11, the non-segmentable feature search conditions in Experiment 4 involve some very small target-distractor (TD) distances within either feature space alone. In contrast, because color and orientation of distractors are negatively correlated in the non-segmentable conjunction case, a small color TD difference will be accompanied by a large orientation difference and vice versa. In a standard segmentable situation, the TD differences in the feature search tasks would be large, resulting in very efficient feature search with slopes near 0. This would be more efficient than guided conjunction search.

It is also important to note, that our finding is more consistent with the simultaneous account of top-down feature guidance (Wolfe, 1994; Wolfe et al., 1989) than with the sequential account (Huang & Pashler, 2007; Zohary & Hochstein, 1989) because it is hard to imagine that the sequential combination of two inefficient feature searches would be efficient.

## Experiment 5

Our failure to find effects of segmentability on search guidance in the earlier experiments is a form of a negative result. It could be objected that we just did not look hard enough. Thus, in a final Experiment 5, we address that possibility. First, in our previous experiments, we used standard samples of ∼12 observers that still could be insufficient to detect modest differences in search efficiency between different segmentability conditions. Here, we double the observer population to 24. Second, one could argue that it is hard to find a difference in slopes in experiments using only two, relatively small set sizes (9 and 17) because grouping/texture effects can be more pronounced with larger, denser arrays of items. Accordingly, we expanded the list of set sizes to include sets of 33 and 65 elements.

### Method

#### Participants

In total, 26 students at the HSE University participated in Experiment 5 for extra course credits (21 female, mean age 19.7). The results of two participants were excluded from the analysis because of an error rate of more than 20%. The chosen sample size (24 observers) allows us to detect medium effect sizes (η^2^ ∼ .2) with a power (1 − β) = 0.85 and an α-level = 0.05. No participants reported any experience of neurological problems and all had normal or corrected-to-normal visual acuity and no evidence of color blindness. At the beginning of experiment, they gave a written informed consent.

#### Apparatus, stimuli, and procedure

**Figure 12.**
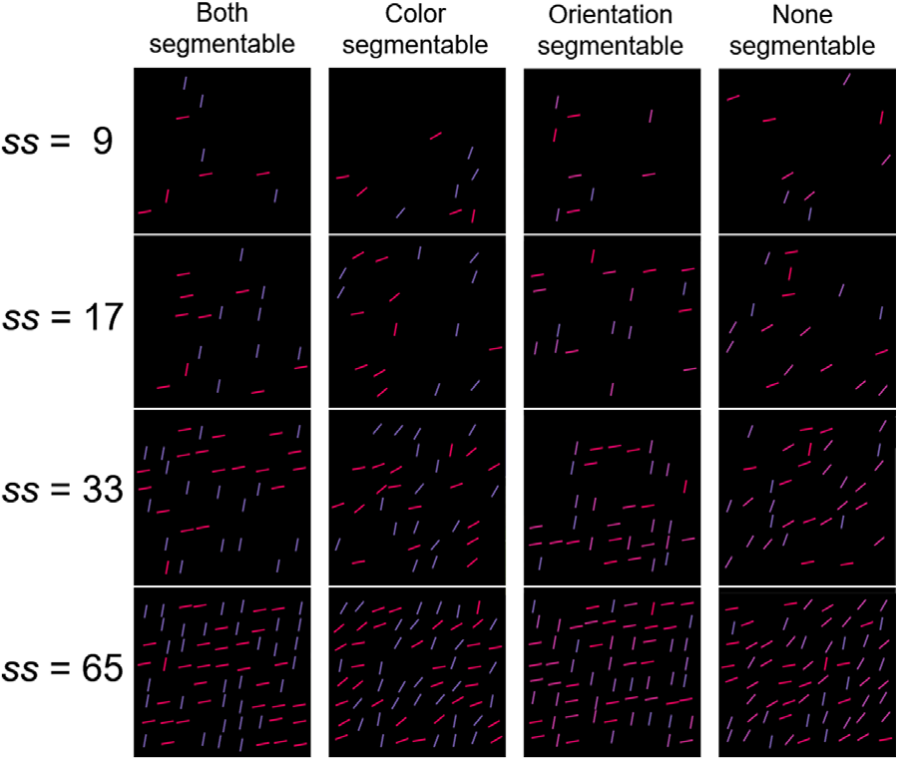
Examples of four segmentability conditions in Experiment 5 for target present trials. Target = red steep line. The lines are slightly enlarged for illustrative purposes. ss, set size.

We used the same apparatus as in Experiment 2. Stimuli and procedure were identical to Experiment 1, except for two changes. First, we used four set sizes. Each set size was provided by presenting the same set of 8 distractors repeated a different number of times: 1×8 distractors + 1 target/distractor = 9 items; 2×8 distractors + 1 target/distractor = 17 items; 4×8 distractors + 1 target/distractor = 33 items; and 8×8 distractors + 1 target/distractor = 65 items. Secondly, as the maximum number of elements on the screen increased, we changed the grid for presenting stimuli. A 28.86° × 25.77° rectangle at the center of the screen was used as the “working” field in this experiment. It was divided into 8 (vertical) × 9 (horizontal) = 72 cells by an imaginary grid (each cell side was 3.28°). Each cell could be used for positioning a single line element of the display (some cells could be empty in a trial). Within a cell, lines were randomly jittered within a ±0.61° range in both horizontal and vertical directions. Figure 12 shows examples for all the types of displays.

#### Design and data analysis

In this experiment, we used a 4 (distractor distribution: both, none, orientation, color) × 2 (target: present vs. absent) × 4 (set size: 9, 17, 33, 65) within-subject design. Fifty trials were presented within each cell of this factorial combination, so the total number of trials was 1600 per observer. The dependent variables and their analyses were the same as in Experiment 1.

### Results

**Figure 13.**
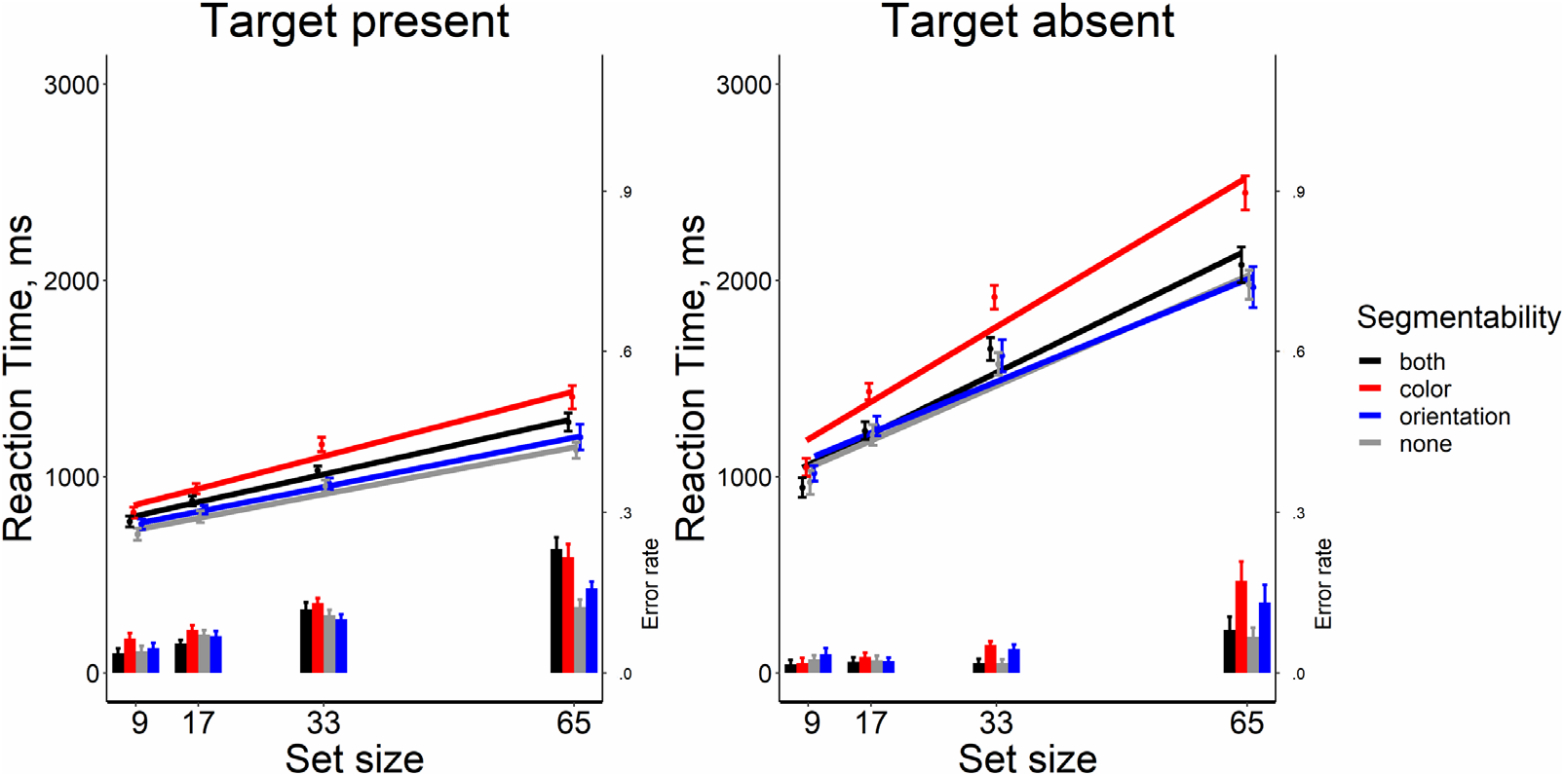
Reaction time and error rate as a function of set size and segmentability in Experiment 5. *Error bars* denote the *SEM,* with between-subject variance removed in accordance with the Cousineau (2005) method.

Figure 13 shows that Experiment 5 replicated the pattern of results in Experiment 1. Overall, the slopes of RT × set size functions were indicative of a relatively efficient search comparable to standard conjunction search results and to our earlier results. On the target-present trials, the slopes were 7-10 ms/item, the target-absent trials showed the search rate of 16 to 20 ms/item. The repeated-measures ANOVA on slopes showed a small effect of distractor segmentability in target-present trials (*F*[3, 69] = 3.599, *p* = 0.018, η^2^ = 0.135, BF_10_ = 2.587). Note that the effect goes in the “wrong” direction. The non-segmentable condition is marginally easier than the color segmentable condition. Pairwise comparisons did not show differences that were significant once correct for multiple comparison (*t*s[23] < 2.417, *p*s > .023, Bonferroni corrected α = 0.008, Cohen's *d*s < 0.494, BF_10_s < 2.341), except that the slopes in the “color segmentable” condition were greater than in the “none segmentable” condition; again the “wrong” direction (*t*[23] = 3.081, *p* = 0.005, Bonferroni corrected α = 0.008, Cohen's *d* = 0.629, BF_10_ = 8.318). For target-absent trials, ANOVA showed an effect of distractor segmentability (*F*[3, 69] = 8.421, *p* < 0.001, η^2^ = 0.268, BF_10_ = 315.243) again, driven by poorer performance in the “color segmentable” condition: slopes in this condition were significantly larger than in the rest conditions (*t*s[29] > 3.014, *p*s < 0.007, Bonferroni corrected α = 0.008, Cohen's *d*s < 0.614, BF_10_s > 7.282), whereas three other conditions did not differ from each other (*t*s[29] < 1.739, *p*s > 0.095, Bonferroni corrected α = 0.008, Cohen's *d*s < 0.356, BF_10_s < 0.79).

We found similar effects of distractor segmentability both on average RTs and error rates for target-present and target-absent trials (*F*[3, 69] > 4.426, *p* < 0.008, η^2^ > 0.16, BF_10_ > 6.01). Pairwise comparisons showed that all effects produced by the fact that participants had more errors and greater RTs in the “color segmentable” condition. This condition produced more difficulties compared to the “none segmentable” one for all these four ANOVAs; also, participants had more errors in the “color segmentable” condition compared to the “orientation segmentable” in target-present trials and greater average RTs compared to “both segmentable” in target-absent trials (*t*s[29] > 3.102, *p*s < 0.006, Bonferroni corrected α = 0.008, Cohen's *d*s > 0.632, BF_10_s > 4.97). Whereas all other conditions had similar values for both parameters (*t*s[29] < 2.858, *p*s > .008, Bonferroni corrected α = 0.008, Cohen's *d*s < 0.584, BF_10_s < 5.332). Also, it is noteworthy that our observers showed some speed-accuracy trade-off (Figure 13) as error rates increase with set size growth.

The main result of Experiment 5 is a successful replication of our principal findings from Experiments 1 to 4. With greater statistical power and a greater range of set sizes (including dense, texture-like displays), we again found that all conjunction searches were quite efficient, and distractor segmentability had no deleterious effect on the efficiency of conjunction search (especially in more diagnostic condition with a target present on the screen). As before, this result suggests that visual system is able to implement simultaneous guidance by two features.

Having said that, under the increased a priori statistical power, we observed one effect probably overlooked in Experiment 1: We found a slight loss in efficiency in the “color segmentable” compared to the “none segmentable” condition in target-present trials. Combined with the similar effects on error rates and average RTs both in target-absent and target-present trials, we can conclude that “color segmentable” displays systematically cause additional difficulty for observers. Interestingly, it was not the “none segmentable” condition that turned out to be the most difficult one. This finding can be explained by the role of target-distractor similarity (Duncan & Humphreys, 1989), although it does not strongly affect our main conclusion. In fact, conditions with only one segmentable feature had some distractors looking more like a target than any distractor from “both segmentable” or “none segmentable” conditions. When we had only two color shades in a “color segmentable” display, some of the distractors sharing color with a target inevitably became similar to a target in terms of orientation, because the whole range of orientations (from 10˚ to 80˚) should be present on the screen. For example, in Figure 12 (set size = 9, “color segmentable” condition), red distractors with 30˚ and 40˚ orientations are very similar to a target (a red 80˚-oriented line). Unlike conditions with only one segmentable feature, all distractors in “both segmentable” and “none segmentable” conditions have roughly the same “feature distance” from a target. It is achieved by full counter-correlation of features: the more a distractor looks like a target along one dimension, the more dissimilar to a target it looks along the other dimension. Interestingly, only the “color segmentable” condition and not the “orientation segmentable” produced this additional search difficulty. This might be explained by the fact that global “color filters” are very finely tuned (Sun, Chubb, Wright & Sperling, 2016), unlike filters for other dimensions, for example orientation (Foster & Ward, 1991; Inverso, Sun, Chubb, Wright & Sperling, 2016). Therefore a tiny difference in color between a target and a distractor of the same orientation (in “orientation segmentable” condition) is enough to produce a quite large target-distractor dissimilarity.

To sum up, the results of Experiment 5 replicate the main findings from the previous experiments, with an addition of the newly found slight effect of the “color segmentable” condition. Our basic pattern of results across all experiments remains well in line with the expectations of the Guided Search model (Wolfe, 1994; Wolfe et al., 1989), suggesting simultaneous guidance by two features, and offering less support to sequential guidance models, such as Boolean Maps theory (Huang & Pashler, 2007).

## General discussion

In our experiments, we tested whether an ability to coarsely categorize and segment items based on their features is a requirement for efficient conjunction search amongst these items. We manipulated the distributional statistics of the features making the items either segmentable (extremely different features, no intermediates between them), or non-segmentable (extreme features were interspersed with more intermediate, “transition” feature values). This “segmentability” property of multi-item displays has been shown to influence the performance of various visual tasks like the texture discrimination task (Utochkin et al., 2018) or visual search (Utochkin & Yurevich, 2016). Here we asked whether segmentability would influence conjunction search. We found that the efficiency of conjunction search, as estimated by the slopes of RT × set size functions, was not strongly affected by the segmentability of distractors. This finding was replicated across all experiments and across two different pairs of tested feature conjunctions, color-orientation (Experiments 1, 3, 4, and 5) and length-orientation (Experiment 2). Interestingly, the efficiency of conjunction search was robust against low segmentability even though basic feature search became less efficient and more error-prone, using the same stimuli (Experiment 4).

This finding sheds light on the role of grouping and categorization in conjunction search. In classic conjunction search, it can seem intuitively clear that grouping and categorization are involved, as they are involved in feature searches (Avraham et al., 2008; Bacon & Egeth, 1994; Palmer et al., 2000; Rosenholtz, 1999; Utochkin & Yurevich, 2016; Wolfe et al., 1992). Intuition follows the logic of the sequential model (e.g. Huang & Pashler, 2007). Conjunction search can certainly feel like a sequence of feature-based operations. If observers search for a red vertical line among red horizontals and green verticals, they will often claim, if asked, that they found the targets by looking for odd items in the red group (e.g. Nakayama & Martini, 2011, but see Friedman-Hill & Wolfe, 1995). A similar devotion to a grouping account is characteristic of many studies in the literature (e.g., Anderson et al., 2012; Driver et al., 1992; Grossberg et al., 1994; Pashler, 1987; Wang et al., 2005; Zohary & Hochstein, 1989). However, there is no reason why guided search to a conjunction target has to involve grouping in each particular feature dimension. Simply guiding to redness and to verticalness (or “steepness”) simultaneously will bias attention toward any red vertical items in the display regardless of whether the red and/or the vertical items form a salient, categorical group. The present results suggest that the impression of a role for categorical grouping in standard conjunction search is an epiphenomenon. Attention is guided to a conjunction by color and orientation (or length and orientation). Color and orientation also support grouping and categorization but these two facts need not to be related. This could be seen as evidence that “effortless” texture segmentation and “parallel” visual search are not the same thing (Wolfe et al., 1992).

The role of grouping in conjunction search, beyond being an interesting research question by itself, was a critical test to differentiate between two kinds of models of top-down feature guidance: simultaneous versus. sequential. If observers had used a sequential strategy, we would have expected damaging effects when a feature was not segmentable. Given an increase in feature heterogeneity and a fuzzier border between distractor groups in non-segmentable displays, conjunction search would seemingly have to become less efficient in those non-segmentable displays. Our data show that this prediction is not the case. Moreover, when participants knew only a target color in advance and so they had to select a color subset first, then to determine what the target orientation would be (Experiment 3), we obtained a quite different pattern of results compared to all other experiments: Here, segmentability had a substantial effect on the absolute search time. The fact that conjunction search can be overall faster and more efficient than its component feature searches (Experiment 4) seems to be problematic for the theories proposing the strictly sequential character of guidance. It is hard to imagine how the sequential combination of two inefficient searches can be efficient. Therefore, we can conclude that it is very unlikely that our participants used sequential feature guidance (as implied by some previous work: Huang & Pashler, 2007; Pashler, 1987; Zohary & Hochstein, 1989). Our results are more consistent with the simultaneous version of guidance, i.e., the Guided Search model (Wolfe, 1994; Wolfe et al., 1989). Guided Search assumes that the knowledge of target features can be used to set priorities of attending locations in the visual field. Such a guidance is provided in a top-down manner by adding activation to all locations containing target features. Borrowing an idea from Treisman's Feature Integration Theory, Guided Search assumes that preattentive feature maps process different features separately and are blind to other feature maps (Treisman & Gelade, 1980). Guided search supposes that the information from all these maps can be accessed at the same time and that a weighted sum (Liesefeld & Müller, 2019) of their activations creates the priority map that guides the deployment of attention. If the target is a conjunction of two or more features, its location gets an advantage over other locations because its total activation would be greater than the activation of other locations containing distractors having only one or neither of the target features. In the Interim discussion, we provided an account of how segmentable and non-segmentable feature distributions would produce very different feature maps while yielding very similar attention-guiding priority maps (Figure 6). This is consistent with the conjunction search results presented here. Also, models of the Guided Search flavor are consistent with the surprising finding that conjunction search can be more efficient than feature searches involving the same, non-segmentable feature distributions. The efficiency of feature search can be diminished by distractors’ heterogeneity in the non-segmentable case while the efficiency of the conjunction search survives because the low TD difference in one feature map is offset by a large difference in the other map (Experiment 4).

Note that we do not argue against sequential models of guidance in general. We think that these simultaneous and sequential modes of guidance can flexibly coexist depending on task requirements. When a task is to find an odd conjunction target without any prior knowledge about its identity, observers may rely more on a sequential guidance strategy. Also, the search for multiple targets (Huang & Pashler, 2012) and texture segmentation (Utochkin et al., 2018) were shown to be performed in a sequential manner. However, when observers have a reliable knowledge of target features, they can use simultaneous guidance by several features. Therefore, sequential guidance is a powerful tool for performing various search tasks but still it is only one of the options; and, certainly, it is not mandatory.

The current results clarify one aspect of feature guidance in guided search. The similarity of segmentable and non-segmentable conjunction search suggests that attention can be guided to “reddish” (or “bluish”) items and not only to a single, precise color of red. This can be seen a similar in spirit to Stefanie Becker's relational account of search (Becker, 2010) in which she argues that attention is better described as being guided to the color that is the “reddest” even if it is not precisely “red.” As Experiment 4 showed, this can impose difficulties for feature search when one needs to find a target with very finely specified features (find exactly that red thing among other reddish things) and when the category is not very distinct (purple things can be equally categorized as reddish and bluish). But this coarse categorization is sufficient to guide conjunction search. This has an important implication for search in the real world. In many situations, target features can be not categorically distinct from distractors, so that the target would not pop out and a search guided by the knowledge of a single feature would proceed inefficiently. However, even the coarse top-down knowledge of several features can guide attention more efficiently and make conjunction search robust against categorical uncertainty of the environment.

In sum, in our experiments, we tested how conjunction search is carried out among highly heterogeneous items with fuzzy transitions between features forming relevant and irrelevant extremes. For the conditions tested here, observers did not pay a cost for the heterogeneity of the search items and their categorical uncertainty. In the real world, conjunctively defined targets are rarely placed amid two homogeneous groups of distractors. These results suggest how guidance might continue to work under those conditions. Of course, grouping by basic features remains important for multiple aspects of everyday vision. However, it does not appear to be a prerequisite for conjunction search.
